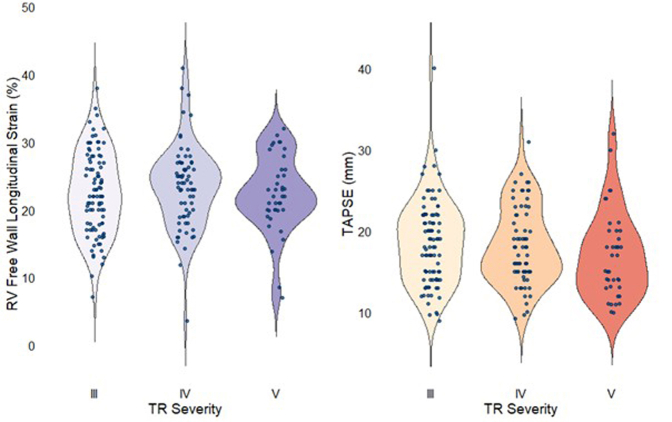# Comparison of Echocardiographic Right Ventricular–Pulmonary Arterial Coupling Indices in Patients Undergoing Transcatheter Tricuspid Valve Repair

**DOI:** 10.1016/j.shj.2026.100801

**Published:** 2026-01-21

**Authors:** Jennifer von Stein, Philipp von Stein, Lukas Stolz, Michael I. Brener, Muhammed Gerçek, Felix Rudolph, Laura Marx, Maria Isabel Körber, Stephan Baldus, Volker Rudolph, Juan F. Granada, Jörg Hausleiter, Roman Pfister, Christos Iliadis

**Affiliations:** a University of Cologne, Faculty of Medicine and University Hospital Cologne, Department III of Internal Medicine, Cologne, Germany; b Clinical Trials Center, Cardiovascular Research Foundation, New York, New York; c Center for Cardiovascular Medicine ABCD, Aachen – Bonn – Cologne – Düsseldorf, Germany; d Medizinische Klinik und Poliklinik I, LMU Klinikum, LMU München, Munich, Germany; e German Center for Cardiovascular Research (DZHK), Partner Site Munich Heart Alliance, Munich, Germany; f Department of Cardiology, Columbia University Irving Medical Center, New York, New York; g Clinic for General and Interventional Cardiology/Angiology, Herz- und Diabeteszentrum NRW, Ruhr-Universität Bochum, Bad Oeynhausen, Germany

**Keywords:** Right ventricular free wall longitudinal strain, RV-PA coupling, Strain analysis, TAPSE/PASP, Transcatheter tricuspid valve repair, Tricuspid regurgitation

## Abstract

**Background:**

Tricuspid annular plane systolic excursion (TAPSE) to pulmonary artery systolic pressure (PASP) is an established prognostic marker in tricuspid regurgitation (TR). Right ventricular free wall longitudinal strain (RVFWS)/PASP has been proposed as more sensitive prognostic markers than TAPSE/PASP in patients with TR, yet its role in patients undergoing transcatheter tricuspid valve repair (TTVr) remains unclear. The objective of the study was to evaluate and compare the prognostic value of RVFWS/PASP and TAPSE/PASP with two different vendors in symptomatic TTVr cohorts.

**Methods:**

TTVr-treated patients across three centers (2017-2023) were included. RVFWS was assessed using two vendors (A = GE EchoPAC and B = Tomtec). The primary endpoint was a composite of all-cause mortality or the first heart failure hospitalization at 2-years. Prognostic performance was assessed using Cox regression, C-statistics, and Kaplan–Meier analysis.

**Results:**

A total of 349 patients were included (137 vendor A and 212 vendor B). Patients in vendor B were older (83 vs. 80; *p* < 0.001), more symptomatic (New York Heart Association III/IV: 95.8 vs. 81.7%; *p* < 0.001), had lower 6-minute walk distance (218 vs. 282 m; *p* < 0.001), and greater right heart dilation, despite similar TR severity. RVFWS was higher in vendor B (23.0 vs. 18.0%, *p* < 0.001). RVFWS/PASP emerged as an independent predictor of the primary endpoint only in vendor A (hazard ratio per %/mmHg increase: 0.07, 95% CI: 0.01–0.46; *p* = 0.005), whereas TAPSE/PASP consistently showed independent prognostic association in both cohorts. Discrimination of both indices was not significantly different. Receiver operating characteristics–derived cutoffs identified patient subgroups with markedly different 2-year outcomes in both cohorts.

**Conclusions:**

Both RVFWS/PASP and TAPSE/PASP provided modest prognostic value after TTVr, with TAPSE/PASP demonstrating consistent independent association across vendors.

## Highlights

• Right ventricle–pulmonary arterial coupling was evaluated using right ventricular free wall longitudinal strain (RVFWS)/pulmonary artery systolic pressure (PASP) and tricuspid annular plane systolic excursion /PASP, assessed with two different vendors in two different cohorts undergoing transcatheter tricuspid valve repair.
• Tricuspid annular plane systolic excursion /PASP emerged as a more robust coupling index, being independently associated with long-term outcomes after transcatheter tricuspid valve repair, whereas RVFWS/PASP showed an association with outcome only in 1 patient cohort, with pronounced intervendor variability in absolute RVFWS values.
• Dichotomous risk stratification based on receiver operating characteristics–derived cutoffs demonstrated a significantly better outcome in patients with higher right ventricle–pulmonary artery coupling in both cohorts.

## Graphical abstract

Comparison of RVFWS/PASP and TAPSE/PASP in predicting 2-year mortality after transcatheter tricuspid valve repair. (A) Representative echocardiogram in a patient with severe tricuspid regurgitation. (B) TAPSE measurement (red arrow) from an M-mode tracing in systole. (C)RVFWS measurement with dedicated software; the region of interest is highlighted by three colored dots, representing the different free-wall segments. (D) Measurement of the peak tricuspid regurgitation velocity (TR Vmax). (E) Respiratory variability of the inferior vena cava, used to estimate the right atrial pressure (RAP). Abbreviations: PASP, estimated pulmonary artery systolic pressure; RV, right ventricle; RVFWS, right ventricular free wall longitudinal strain; TAPSE, tricuspid annular plane systolic excursion; TR, tricuspid regurgitation.

**Figure:**
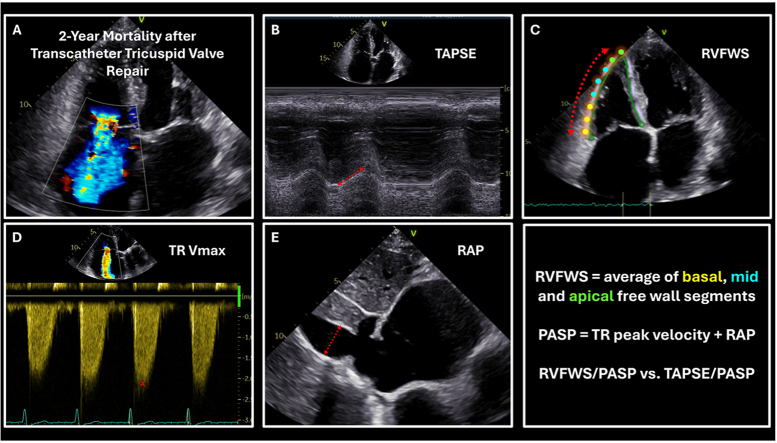

## Introduction

Untreated severe tricuspid regurgitation (TR) is associated with poor prognosis as it exposes the right ventricle (RV) to volume overload, leading to RV dysfunction, and clinical heart failure.^1^ Recognition of these deleterious effects of TR has led to the development and implementation of a variety of options for transcatheter tricuspid valve repair (TTVr). However, reliable prognostic stratification in this population remains a major clinical challenge.

RV-pulmonary arterial (RV-PA) coupling—typically estimated by the ratio of tricuspid annular plane systolic excursion (TAPSE) to PA systolic pressure (PASP)—has been associated with clinical outcomes in patients with TR.^2^^,^^3^ However, given the load dependency of TAPSE, it may overestimate RV systolic function in the setting of severe TR, raising concerns about its reliability in this context.^4^

In contrast, speckle-tracking echocardiography enables a less angle- and load-dependent assessment of longitudinal myocardial performance.^5^ Accordingly, RV free wall longitudinal strain (RVFWS) has emerged as a sensitive parameter for detecting early RV dysfunction and demonstrated incremental prognostic value over RV fractional area change (FAC) and TAPSE in patients with severe TR.^6^

Although RVFWS/PASP has previously been linked to adverse outcomes in patients with severe secondary mitral regurgitation enrolled in the COAPT trial^7^ and conservatively managed patients with severe TR,^8^ its prognostic value in patients undergoing TTVr remains uncertain, particularly in comparison with TAPSE/PASP. In addition, strain values are known to be vendor-dependent, which limits their comparability across different platforms.^5^^,^^9^

Currently, data on RVFWS-derived RV-PA coupling in patients undergoing TTVr are lacking. Therefore, the aim of this study was to compare the discriminatory power of these two echocardiographic RV-PA coupling indices and their prognostic performance across two vendor-defined cohorts.

## Methods

### Study Design and Population

**Supplementary Figure 1:**
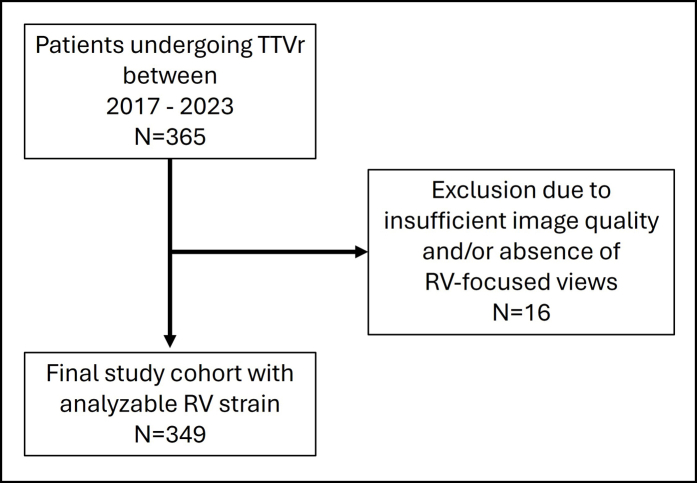


This multicenter, retrospective study included consecutive patients with symptomatic TR who underwent TTVr between 2017 and 2023 at three high-volume tertiary German centers (University Heart Centers in Cologne, Munich, and Bad Oeynhausen). The derivation of the final study cohort is shown in Supplementary Figure 1. All patients underwent evaluation by a local multidisciplinary heart team before TTVr. Institutional review board approval was obtained at all participating sites, and the requirement for written informed consent was waived due to the retrospective nature of the analysis.

Clinical follow-up assessments, performed by prescheduled ambulatory visits as part of routine clinical practice, included New York Heart Association (NYHA) class, hospitalizations for heart failure (HFH), and survival status. For patients unable to return to the intervention center, follow-up was conducted via telephone. Outcomes were based on the Tricuspid Valve Academic Research Consortium definitions.^10^

TTVr was performed either via tricuspid valve transcatheter edge-to-edge repair (T-TEER) using the TriClip (Abbott Vascular, Abbott Park, Illinois) or the PASCAL (Edwards Lifesciences, Irvine, California), or via direct transcatheter tricuspid valve annuloplasty (TTVA) using the Cardioband (Edwards Lifesciences, Irvine, California). The choice between T-TEER and TTVA was based on anatomical considerations (i.e., leaflet morphology/geometry, extent of coaptation gap and jet location), as previously described.^11^

Patients were stratified into two cohorts according to the vendor-specific software used for RV strain quantification: RV strain in the vendor A cohort was assessed using GE EchoPAC (GE Healthcare, Chicago, Illinois), and in the vendor B cohort using Tomtec Image Arena (Tomtec Imaging Systems GmbH, Unterschleißheim, Germany).

### Echocardiographic Assessment

All patients underwent comprehensive transthoracic echocardiography before intervention. Images were stored digitally and analyzed offline by experienced imaging specialists at each center according to current guideline recommendations.^12^

TAPSE was calculated from M-mode recordings of the lateral tricuspid annulus and RV end-systolic and end-diastolic areas were traced in the RV-focused view to calculate the RVFAC. PASP was derived from the peak transvalvular TR velocity, and the semiquantitative approximated right atrial pressure depending on the size and variability of the inferior vena cava.

From an RV-focused apical four-chamber view, the RV free wall was manually traced at end systole. The region of interest was adjusted to include the full myocardial thickness. The RV free wall was divided into basal, mid, and apical segments, and RVFWS was calculated as the average of these three segments. All strain values are presented as absolute values. Impaired RVFWS was defined as a value < 20% for both cohorts.^10^^,^^13^ RV-PA coupling was assessed using the ratio of RVFWS or TAPSE to estimated PASP (PASP). Invasively measured PASP (PASP_inv_) was reported for patients with available hemodynamic data. TR severity was assessed using a multiparametric approach and categorized into the following grades: none (0), mild (I), moderate (II), severe (III), massive (IV), and torrential (V).^14^ Atrial and ventricular phenotypes were classified based on the presence or absence of a preserved left ventricular ejection fraction (>50%), preserved RV longitudinal function (TAPSE >17 mm), and an end-systolic right-atrial–to–right-ventricular area ratio (>1.5).^15^

### Study Endpoints and Outcomes

The primary endpoint was survival free from HFH at 2 years. Secondary endpoints were the change in NYHA class (≥1 class) and TR grade compared with baseline, assessed at the latest available follow-up after discharge.

### Statistical Analysis

Continuous variables are reported as mean ± SD or as median with interquartile range (Q1-Q3), as appropriate. Categorical variables are presented as counts and percentages. Normality was assessed using the Kolmogorov–Smirnov test. Group comparisons were performed using the χ^2^ or Fisher exact test for categorical variables, and the Student’s t-test or Mann–Whitney U test for continuous variables, as appropriate. Paired analyses were performed using the Wilcoxon signed-rank test.

All subsequent analyses were conducted separately for vendor A and vendor B to account for vendor-specific variability in strain measurements. Clinical and echocardiographic baseline characteristics were compared between the two cohorts.

The primary endpoint, the composite of survival free from HFH, was analyzed as a time-to-first-event. The discriminatory ability of RVFWS/PASP and TAPSE/PASP was quantified using the Harrell concordance index (C-statistic) with 95% CIs derived from SEs and compared using an approximate z-test.

Univariable and multivariable Cox proportional hazards models were performed to evaluate the association of RVFWS/PASP and TAPSE/PASP with the primary endpoint in separate models. The proportional hazards assumption was verified using Schoenfeld residuals, with no significant violations observed. Multivariable models included parameters with univariable significance of *p* < 0.05; RVFWS/PASP or TAPSE/PASP were forced into the models based on clinical and conceptual relevance. Before model entry, all covariates were assessed for multicollinearity using variance inflation factors and pairwise correlation matrices, with a correlation coefficient threshold of r > 0.7. Results are reported as hazard ratios (HRs) with corresponding 95% CIs and *p* values.

Receiver operating characteristics (ROC) analyses were performed to examine the association of RVFWS/PASP and TAPSE/PASP with the primary endpoint. Optimal thresholds were determined by the Youden index, and vendor-specific cohorts were stratified into high (>threshold) and low (≤threshold) groups accordingly. Kaplan–Meier curves were used to estimate survival free from HFH, and groups were compared using the log-rank test.

Correlation analyses between RVFWS or TAPSE and PASP as well as TR severity were performed using Spearman’s rank correlation coefficient.

Sensitivity analyses were conducted in subgroups stratified by procedure type and in patients with available hemodynamic data. Bland–Altman analysis was applied to assess agreement between estimated and invasively measured PASP.

All analyses were two-tailed, and *p* values < 0.05 were considered statistically significant. Statistical analyses were performed using R (version 4.3.2; R Foundation for Statistical Computing, Vienna, Austria).

## Results

### Baseline Characteristics

A total of 349 patients were included in this analysis, comprising 137 in vendor A and 212 in vendor B. Baseline characteristics according to vendor cohort are summarized in Table 1. Compared with vendor A patients, those in vendor B were older, more frequently males, had a shorter 6-minute walk distance, received higher loop diuretic doses, and more often presented in NYHA class III or IV.

**Table 1:** Baseline and procedural characteristics

Clinical characteristics	Vendor A N = 137	Vendor B N = 212	*p* value
Age, y	80 [74-83]	83 [79-86]	**<0.001**
Body mass index, kg/m^2^	25.5 [22.5-29.3]	24.4 [21.8-27.7]	**0.028**
Female	87 (63.5)	102 (48.1)	**0.006**
EuroSCORE II, %	4.04 [2.8-7.1]	4.38 [2.8-6.6]	0.829
TRI-SCORE	5 [4-6]	6 [4-7]	0.260
New York Heart Association			**<0.001**
II	25 (18.2)	9 (4.2)	
III	94 (68.6)	174 (82.1)	
IV	18 (13.1)	29 (13.7)	
6MWD, m	282 [207-356] (N = 92)	218 [150-310] (N = 192)	**<0.001**
Furosemide equivalent, mg	40 [20-80]	55 [25-100]	**0.004**
Comorbidities			
Arterial hypertension	116 (84.7)	174 (82.1)	0.562
Diabetes mellitus	32 (23.4)	41 (19.3)	0.419
Atrial fibrillation	129 (94.2)	193 (91)	0.313
Coronary artery disease	52 (38.0)	81 (38.2)	>0.999
History of myocardial infarction	9 (6.6)	15 (7.1)	>0.999
History of cardiac surgery	24 (17.5)	52 (24.5)	0.144
History of stroke	11 (8.0)	24 (11.3)	0.419
Pacemaker	33 (24.1)	66 (31.1)	0.181
Chronic lung disease	24 (17.5)	24 (11.3)	0.113
Laboratory data			
eGFR, mL/min	43 [33-54]	38 [28-53]	**0.021**
<60 ml/min	112 (81.8)	169 (79.7)	0.680
On dialysis	1 (0.7)	6 (2.8)	0.253
Bilirubin, mg/dl	0.7 [0.5-1.0]	0.9 [0.6-1.3]	**<0.001**
NT-proBNP, pg/mL	2016 [1276-3296]	1994 [1224-4211]	0.543
Procedural Characteristics			
Transcatheter treatment modality			**<0.001**
T-TEER	80 (58.4)	212 (100)	
TTVA	57 (41.6)	0 (0.0)	

Notes. Values are n (%) or median [Q1-Q3]. Bold values indicate statistical significance.

Abbreviations: 6MWD, 6-min walk distance; eGFR, estimated glomerular filtration rate; EuroSCORE II, European System for Cardiac Operative Risk Evaluation II; NT-proBNP, N-terminal pro–B-type natriuretic peptide; T-TEER, tricuspid valve transcatheter edge-to-edge repair; TTVA, transcatheter tricuspid valve annuloplasty.

Comorbidities were generally similar between cohorts, including the prevalence of arterial hypertension, coronary artery disease, and atrial fibrillation. Surgical risk, as assessed by EuroSCORE II and TRI-SCORE, did not differ between groups, nor did N-terminal pro–B-type natriuretic peptide levels.

In vendor B, all patients underwent T-TEER, whereas in vendor A 58.4% were treated with T-TEER and 41.6% with TTVA.

### Echocardiographic Baseline Assessment

Echocardiographic baseline characteristics by vendor cohort are summarized in Table 2. Patients in vendor B had significantly larger right heart dimensions compared with vendor A. TAPSE and RVFAC were similar between groups, whereas RVFWS and right ventricular global longitudinal strain were significantly lower in vendor A. In addition, PASP was higher in vendor A, and consequently RV-PA coupling indices for both RVFWS/PASP (0.42 [0.30–0.58] vs. 0.59 [0.49–0.79], *p* < 0.001) and TAPSE/PASP (0.39 [0.33–0.51] vs. 0.45 [0.37–0.61], *p* = 0.001) were lower in this cohort. The distribution of TR grades did not differ significantly between cohorts.

**Table 2:** Echocardiographic assessment at baseline

	Vendor A N = 137	Vendor B N = 212	*p* value
Right atrial area, cm^2^	33.2 [28.0-41.1]	37.0 [30.2-47.2]	**0.003**
RV basal diameter, mm	45.0 [41.0-49.1]	47.0 [41.0-53.0]	**0.030**
RV end diastolic area, cm^2^	20.6 [16.7-27.3]	27.9 [22.0-34.1]	**<0.001**
RV end systolic area, cm^2^	13.0 [10.5-17.0]	17.2 [14.0-21.0]	**<0.001**
RVFAC, %	36.0 [29.4-44.0]	37.5 [31.3-44.4]	0.293
TAPSE, mm	18 [15-20]	18 [14-22]	0.872
RVFWS, %	18.0 [15.0-23.0]	23.0 [19.0-27.0]	**<0.001**
RVGLS, %	15.5 [13.0-19.1]	19.8 [16.3-23.2]	**<0.001**
PASP, mmHg	43 [34-52]	36 [30-46]	**<0.001**
PASP_inv_, mmHg	42 [36-50] N = 103	44.5 [37-56] N = 168	0.118
RVFWS/PASP, %/mmHg	0.42 [0.3-0.58]	0.59 [0.49-0.79]	**<0.001**
TAPSE/PASP, mm/mmHg	0.39 [0.33-0.51]	0.45 [0.37-0.61]	**0.001**
Tricuspid regurgitation			
Vena contracta width, mm	12.0 [9.0-16.0]	9.9 [7.9-12.1]	**<0.001**
Effective regurgitant orifice area, cm^2^	0.54 [0.42-0.77]	0.50 [0.38-0.70]	**0.008**
Regurgitant volume, mL	46.0 [36.5-60.0]	38.0 [29.0-50.0]	**<0.001**
Tricuspid regurgitation grade			0.054
I	0 (0.0)	0 (0.0)	
II	0 (0.0)	8 (3.8)	
III	71 (51.8)	93 (43.9)	
IV	37 (27.0)	71 (33.5)	
V	29 (21.2)	40 (18.9)	
Tricuspid regurgitation etiology			0.637
Primary	3 (2.2)	7 (3.3)	
Secondary	128 (93.4)	192 (90.6)	
Atrial	49 (38.3)	73 (38.0)	>0.999
Ventricular	79 (61.7)	119 (62.0)	>0.999
Lead-associated	6 (4.4)	13 (6.1)	
Left ventricle			
Left ventricular ejection fraction, %	55 [51-60]	56 [48-63]	0.911
LVEDD, mm	46 [41-51]	47 [43-53]	0.067
Mitral regurgitation grade			**0.010**
0	8 (5.8)	19 (9)	
1+	75 (54.7)	141 (66.5)	
2+	47 (34.3)	46 (21.7)	
3+	2 (1.5)	5 (2.4)	
4+	5 (3.6)	1 (0.5)	

Notes. Values are n (%) or median [Q1-Q3]. Bold values indicate statistical significance.

Abbreviations: LVEDD, eft ventricular end diastolic diameter; PASP, estimated pulmonary artery systolic pressure; PASP_inv_, invasive pulmonary artery systolic pressure; RV, right ventricle; RVGLS, right ventricular global longitudinal strain; RVFAC, right ventricular fractional area change; RVFWS, right ventricular free wall longitudinal strain; TAPSE, tricuspid annular plane systolic excursion.

### Follow-Up and Clinical Outcomes

At 1 year, the Kaplan–Meier estimated incidence of the combined endpoint was 21.3% (95% CI: 14.1%–27.9%) in vendor A and 24.9% (95% CI: 18.8%–30.6%) in vendor B. At 2 years, corresponding event rates were 34.3% (95% CI: 25.2%–42.4%) and 38.0% (95% CI: 30.6%–44.6%).

NYHA class improved significantly in both cohorts (each *p* < 0.001). In vendor A, 60 of 105 patients (57.1%) demonstrated an improvement by ≥ 1 NYHA class at a median of 538 days 3[70–741], whereas in vendor B, 107 of 181 patients (59.1%) improved at a median of 366 days [157–718].

Similarly, among patients with paired echocardiographic follow-up, TR grade improved significantly from baseline to follow-up in both cohorts (each *p* < 0.001). In vendor A, 81 of 106 patients (76.4%) had residual TR ≤ 2+ at the latest available echocardiographic follow-up (median 438 days [213–738]), with 93 (87.7%) improving by ≥ 1 grade and 61 (57.5%) by ≥ 2 grades. In vendor B, 159 of 183 patients (86.9%) showed residual TR ≤ 2+ at follow-up (median 366 days [144–716]), with 171 (93.4%) improving by ≥ 1 grade and 127 (69.4%) by ≥ 2 grades. The proportion of patients with residual TR ≤ 2+ was higher in vendor B than in vendor A (*p* = 0.034).

### Outcomes According to RV-PA Coupling

In vendor A, both RVFWS/PASP and TAPSE/PASP showed modest discrimination for the primary endpoint, with Harrell C-indices of 0.621 (95% CI: 0.537–0.705) and 0.646 (95% CI: 0.566–0.726), respectively. In vendor B, the corresponding C-indices were slightly lower, with 0.564 (95% CI: 0.505–0.623) for RVFWS/PASP and 0.599 (95% CI: 0.543–0.655) for TAPSE/PASP. The difference in discriminative performance between RVFWS/PASP and TAPSE/PASP was not statistically significant in either cohort (vendor A: *p* = 0.673; vendor B: *p* = 0.399).

In vendor A, RVFWS/PASP emerged as an independent predictor of the primary endpoint (adjusted HR: 0.07, 95% CI: 0.01–0.46; *p* = 0.005), alongside female sex (HR: 0.50, 95% CI: 0.28–0.92; *p* = 0.025) and coronary artery disease (HR: 1.92, 95% CI: 1.05–3.51; *p* = 0.033). In contrast, in vendor B, baseline TR severity was the only independent predictor for the primary endpoint (adjusted HR: 1.43, 95% CI: 1.05–1.94; *p* = 0.022) (Tables 3, and 4). Although RVFWS/PASP was independently associated with the endpoint only in vendor A, replacing it with TAPSE/PASP in the models yielded a consistent independent association with outcome in both cohorts (Tables 5 and 6).

**Table 3:** Cox proportional hazards models for 2-y survival free from heart failure hospitalization in vendor A using RVFWS/PASP

Variable	Univariable analysis			Multivariable analysis		
	HR	95% CI	*p* value	HR	95% CI	*p* value
Demographics and clinical characteristics						
Age (per y)	1.00	0.96–1.04	0.986	-	-	-
Sex (female vs. male)	0.49	0.27–0.89	**0.019**	0.50	0.28–0.92	**0.025**
Coronary artery disease	2.26	1.24–4.12	**0.008**	1.92	1.05–3.51	**0.033**
History of myocardial infarction	1.71	0.61–4.79	0.307	-	-	-
Pacemaker	1.63	0.86–3.08	0.136	-	-	-
Echocardiographic parameters						
LVEF (per % increase)	0.98	0.95–1.01	0.123	-	-	-
Baseline MR grade (per grade)	1.25	0.87–1.80	0.232	-	-	-
Baseline TR grade (per grade)	1.05	0.72–1.53	0.789	-	-	-
RVFWS (per % increase)	0.93	0.88–0.99	**0.025**	∗	∗	∗
RVFWS/PASP (per %/mmHg increase)	0.07	0.01–0.39	**0.003**	0.07	0.01–0.46	**0.005**
RV basal diameter (per mm increase)	1.03	0.99–1.08	0.125	-	-	-
Procedural characteristics						
Type of procedure (TTVA vs. T-TEER)	0.82	0.44–1.52	0.531	-	-	-

Notes. Hazard ratios (HRs) and 95% CIs are reported for the association with the primary endpoint at 2 y. Bold values indicate statistical significance.

Abbreviations: HR, hazard ratio; LVEF, left ventricular ejection fraction; MR, mitral regurgitation; PASP, estimated pulmonary artery systolic pressure; RV, right ventricle; RVFWS, right ventricular free wall longitudinal strain; TR, tricuspid regurgitation; T-TEER, tricuspid valve transcatheter edge-to-edge repair; TTVA, transcatheter tricuspid valve annuloplasty.

∗ Not included in the multivariable model due to collinearity with RVFWS/PASP.

**Table 4:** Cox proportional hazards models for 2-y survival free from heart failure hospitalization in vendor B using RVFWS/PASP

Variable	Univariable analysis			Multivariable analysis		
	HR	95% CI	*p* value	HR	95% CI	*p* value
Demographics and clinical characteristics						
Age (per y)	1.03	0.99–1.07	0.117	-	-	-
Sex (female vs. male)	0.62	0.39–0.98	**0.041**	0.67	0.41–1.09	0.110
Coronary artery disease	1.21	0.76–1.92	0.427	-	-	-
History of myocardial infarction	2.27	1.13–4.56	**0.022**	1.84	0.85–3.97	0.120
Pacemaker	1.62	1.01–2.59	**0.045**	1.55	0.95–2.55	0.083
Echocardiographic parameters						
LVEF (per % increase)	0.98	0.96–1.00	**0.015**	0.99	0.97–1.01	0.372
Baseline MR grade (per grade)	1.43	1.02–2.00	**0.040**	1.38	0.99–1.93	0.059
Baseline TR grade (per grade)	1.33	1.02–1.75	**0.037**	1.43	1.05–1.94	**0.022**
RVFWS (per % increase)	0.99	0.95–1.03	0.474	∗	∗	∗
RVFWS/PASP (per %/mmHg increase)	0.72	0.27–1.95	0.520	0.57	0.20–1.67	0.305
RV basal diameter (per mm increase)	1.00	0.97–1.02	0.797	-	-	-

Notes. Hazard ratios (HRs) and 95% CIs are reported for the association with the primary endpoint at 2 y. Bold values indicate statistical significance.

Abbreviations: HR, hazard ratio; LVEF, left ventricular ejection fraction; MR, mitral regurgitation; PASP, estimated pulmonary artery systolic pressure; RV, right ventricle; RVFWS, right ventricular free wall longitudinal strain; TR, tricuspid regurgitation.

∗ Not included in the multivariable model due to collinearity with RVFWS/PASP.

**Table 5:** Cox proportional hazards models for 2-y survival free from heart failure hospitalization in vendor A using TAPSE/PASP

Variable	Univariable analysis			Multivariable analysis		
	HR	95% CI	*p* value	HR	95% CI	*p* value
Demographics and clinical characteristics						
Age (per y)	1.00	0.96–1.04	0.986	-	-	-
Sex (female vs. male)	0.49	0.27–0.89	**0.019**	0.53	0.29-0.96	**0.037**
Coronary artery disease	2.26	1.24–4.12	**0.008**	2.11	1.16-3.85	**0.015**
History of myocardial infarction	1.71	0.61–4.79	0.307	-	-	-
Pacemaker	1.63	0.86–3.08	0.136	-	-	-
Echocardiographic parameters						
LVEF (per % increase)	0.98	0.95–1.01	0.123	-	-	-
Baseline MR grade (per grade)	1.25	0.87–1.80	0.232	-	-	-
Baseline TR grade (per grade)	1.05	0.72–1.53	0.789	-	-	-
TAPSE (per mm increase)	0.92	0.85-0.99	**0.045**	∗	∗	∗
TAPSE/PASP (per mm/mmHg increase)	0.03	0.00-0.28	**0.002**	0.03	0.00-0.31	**0.003**
RV basal diameter (per mm increase)	1.03	0.99–1.08	0.125	-	-	-
Procedural characteristics						
Type of procedure (TTVA vs. T-TEER)	0.82	0.44–1.52	0.531	-	-	-

Notes. Hazard ratios (HRs) and 95% CIs are reported for the association with the primary endpoint at 2 years. Bold values indicate statistical significance.

Abbreviations: HR, hazard ratio; LVEF, left ventricular ejection fraction; MR, mitral regurgitation; PASP, estimated pulmonary artery systolic pressure; RV, right ventricle; TAPSE, tricuspid annular plane systolic excursion; RVFWS,right ventricular free wall longitudinal strain/pulmonary artery systolic pressure; TR, tricuspid regurgitation; T-TEER, tricuspid valve transcatheter edge-to-edge repair; TTVA, transcatheter tricuspid valve annuloplasty.

∗ Not included in the multivariable model due to collinearity with RVFWS/PASP.

**Table 6:** Cox proportional hazards models for 2-y survival free from heart failure hospitalization in vendor B using TAPSE/PASP

Variable	Univariable analysis			Multivariable analysis		
	HR	95% CI	*p* value	HR	95% CI	*p* value
Demographics and clinical characteristics						
Age (per y)	1.03	0.99–1.07	0.117	-	-	-
Sex (female vs. male)	0.62	0.39–0.98	**0.041**	1.01	0.74-1.39	0.941
Coronary artery disease	1.21	0.76–1.92	0.427	-	-	-
History of myocardial infarction	2.27	1.13–4.56	**0.022**	1.44	0.84-2.49	0.189
Pacemaker	1.62	1.01–2.59	**0.045**	1.50	1.06-2.14	**0.023**
Echocardiographic parameters						
LVEF (per % increase)	0.98	0.96–1.00	**0.015**	1.00	0.98-1.01	0.569
Baseline MR grade (per grade)	1.43	1.02–2.00	**0.040**	1.15	0.92-1.44	0.219
Baseline TR grade (per grade)	1.33	1.02–1.75	**0.037**	1.21	0.99-1.48	0.067
TAPSE (per mm increase)	0.92	0.87-0.97	**0.008**	∗	∗	∗
TAPSE/PASP (per mm/mmHg increase)	0.13	0.03-0.53	**0.004**	0.09	0.03-0.26	**<0.001**
RV basal diameter (per mm increase)	1.00	0.97–1.02	0.797	-	-	-

Notes. Hazard ratios (HRs) and 95% CIs are reported for the association with the primary endpoint at 2 years. Bold values indicate statistical significance.

Abbreviations: HR, hazard ratio; LVEF, left ventricular ejection fraction; PASP, estimated pulmonary artery systolic pressure; RV, right ventricle; RVFWS,right ventricular free wall longitudinal strain/pulmonary artery systolic pressure; TAPSE, tricuspid annular plane systolic excursion; TR, tricuspid regurgitation.

∗ Not included in the multivariable model due to collinearity with RVFWS/PASP.

**Figure 1:**
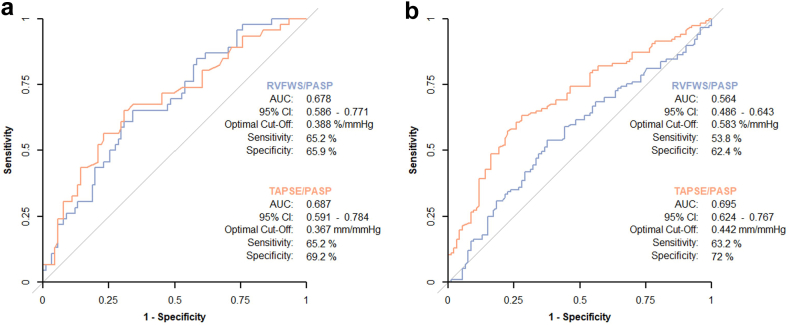
**Receiver operating characteristic analysis of RVFWS/PASP and TAPSE/PASP in vendor-specific cohorts.** ROC curves illustrating the discriminative performance of RVFWS/PASP (blue) and TAPSE/PASP (orange) for predicting the primary endpoint at 2 years in (a) vendor A and (b) vendor B. AUC with 95% confidence intervals, optimal cutoff values, and corresponding sensitivity and specificity are shown. Abbreviations: AUC, area under the curve; PASP, estimated pulmonary artery systolic pressure; ROC, receiver operator characteristic; RVFWS, right ventricular free wall longitudinal strain; TAPSE, tricuspid annular plane systolic excursion.

**Figure 2:**
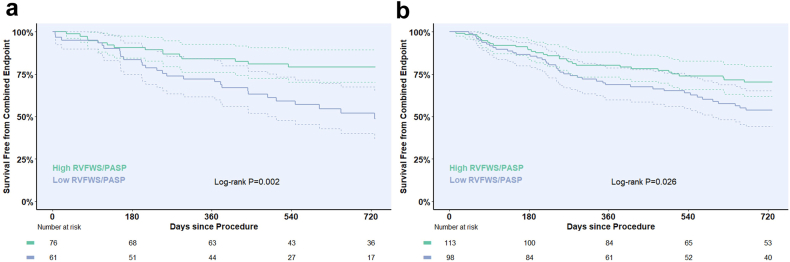
**Kaplan–Meier estimates of 2-year survival free from the combined endpoint by RVFWS/PASP in vendor-specific cohorts.** Kaplan–Meier curves for 2-year survival free from the combined endpoint, stratified by ROC-derived RVFWS/PASP cutoffs in (a) vendor A (cutoff 0.388%/mmHg) and (b) vendor B (cutoff 0.583%/mmHg). In vendor A, patients with high RVFWS/PASP demonstrated significantly higher survival compared with those with low values (79.2% [95% CI: 70.3–89.3] vs. 48.7% [95% CI: 36.4–65.1]; log-rank *p* = 0.002). In vendor B, high RVFWS/PASP was likewise associated with improved survival (70.2% [95% CI: 61.8–79.8] vs. 53.7% [95% CI: 44.2–65.3]; log-rank *p* = 0.026). Abbreviations: PASP, estimated pulmonary artery systolic pressure; ROC, receiver operator characteristic; RVFWS, right ventricular free wall longitudinal strain.

**Figure 3:**
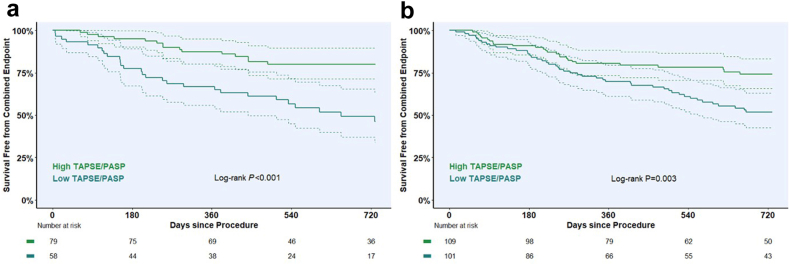
**Kaplan–Meier estimates of 2-year survival free from the combined endpoint by TAPSE/PASP in vendor-specific cohorts.** Kaplan–Meier curves for 2-year survival free from the combined endpoint, stratified by ROC-derived TAPSE/PASP cutoffs in (a) vendor A (cutoff 0.367 mm/mmHg) and (b) vendor B (cutoff 0.442 mm/mmHg). In vendor A, patients with high TAPSE/PASP demonstrated significantly higher survival compared with those with low values (80.0% [95% CI: 71.4–89.7] vs. 46.2% [95% CI: 33.9–63.0]; log-rank *p* < 0.001). In vendor B, high TAPSE/PASP was likewise associated with improved survival (74.1% [95% CI: 65.9–83.4] vs. 51.8% [95% CI: 42.6–63.0]; log-rank *p* = 0.003). Abbreviations: PASP, estimated pulmonary artery systolic pressure; ROC, receiver operator characteristic; TAPSE, tricuspid annular plane systolic excursion.

Kaplan–Meier analysis based on ROC-derived cutoffs for each cohort (Figure 1) demonstrated that patients with a low RVFWS/PASP ratio (<0.388 and < 0.583, respectively) had significantly lower 2-year event-free survival compared to those with high RVFWS/PASP (≥0.388 and ≥ 0.583, respectively), both in vendor A (48.7% [95% CI: 36.4–65.1] vs. 79.2% [95% CI: 70.3–89.3]; *p* = 0.002) and vendor B (53.7% [95% CI: 44.2–65.3] vs. 70.2% [95% CI: 61.8–79.8]; *p* = 0.026) (Figure 2). Similarly, patients with low TAPSE/PASP had markedly impaired 2-year survival in both cohorts (Figure 3).

### Sensitivity Analysis for the Primary Endpoint

Sensitivity analysis stratified by intervention type within the vendor A cohort demonstrated similar discriminative performance for both coupling parameters, with overall higher values in the TTVA subgroup (RVFWS/PASP: 0.693, 95% CI: 0.561–0.825; TAPSE/PASP: 0.672, 95% CI: 0.531–0.814; *p* = 0.834) compared to the T-TEER subgroup (RVFWS/PASP: 0.572, 95% CI: 0.464–0.681; TAPSE/PASP: 0.627, 95% CI: 0.527–0.727; *p* = 0.469).

**Figure 4:**
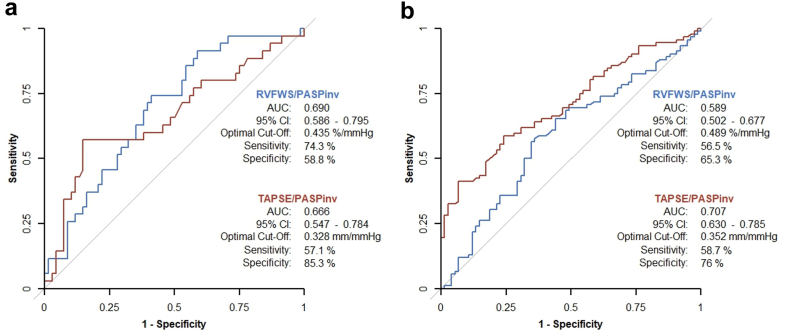
**Receiver operating characteristic analysis of RVFWS/PASP_inv_ and TAPSE/PASP_inv_ in vendor-specific cohorts.** ROC curves illustrating the discriminative performance of RVFWS/PASP_inv_ (blue) and TAPSE/PASP_inv_ (red) for predicting the primary endpoint at 2 years in (a) vendor A and (b) vendor B. AUC with 95% confidence intervals, optimal cutoff values, and corresponding sensitivity and specificity are shown. Abbreviations: AUC, area under the curve; PASP_inv_, invasive pulmonary artery systolic pressure; ROC, receiver operator characteristic; RVFWS, right ventricular free wall longitudinal strain; TAPSE, tricuspid annular plane systolic excursion.

**Supplementary Figure 2:**
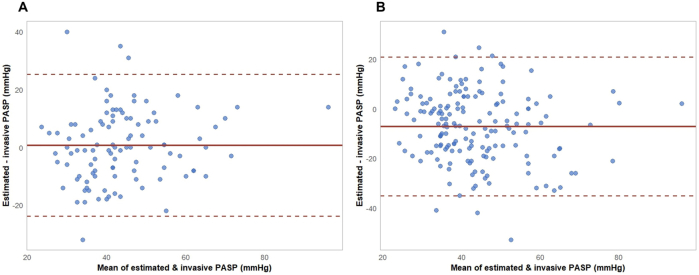


In the subgroups of patients with available hemodynamic data, coupling indices based on echocardiographically estimated PASP showed significant correlations with PASP_inv_ (RVFWS/PASP: *rho* = 0.668, *p* < 0.001; TAPSE/PASP: *rho* = 0.623, *p* < 0.001). Using PASP_inv_, C-indices were numerically higher for both parameters compared to echocardiographic estimates, but remained similar between RVFWS/PASP_inv_ and TAPSE/PASP_inv_ in vendor A (0.637 [95% CI: 0.537–0.736] vs. 0.632 [95% CI: 0.528–0.736]; *p* = 0.952) and vendor B (0.623 [95% CI: 0.558–0.687] vs. 0.633 [95% CI: 0.571–0.695]; *p* = 0.823). Corresponding ROC curves are presented in Figure 4. Agreement between echocardiographic and invasive PASP measurements is shown in Supplementary Figure 2, demonstrating a minimal bias in vendor A and a negative bias with wider limits of agreement in vendor B.

### Association of RVFWS and TAPSE with PASP and TR Severity

**Figure 5:**
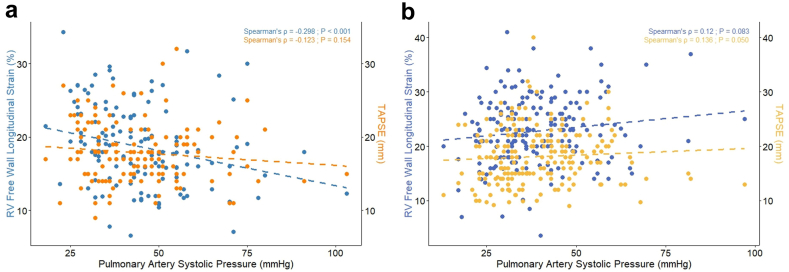
**Correlation of RVFWS and TAPSE with estimated PASP in vendor-specific cohorts.** Scatterplots showing the associations of RVFWS (blue; left Y-axis) and TAPSE (orange; right Y-axis) with estimated PASP. (a) In vendor A, RVFWS demonstrated a modest inverse correlation with PASP (ρ =–0.298, *p* < 0.001), whereas TAPSE showed no significant association (ρ = –0.123, *p* = 0.154). (b) In vendor B, neither RVFWS (ρ =–0.12, *p* = 0.083) nor TAPSE (ρ = 0.136, *p* = 0.050) showed a significant correlation with PASP. Abbreviations: PASP, estimated pulmonary artery systolic pressure; RV, right ventricle; RVFWS, right ventricular free wall longitudinal strain; TAPSE, tricuspid annular plane systolic excursion.

**Supplementary Figure 3:**
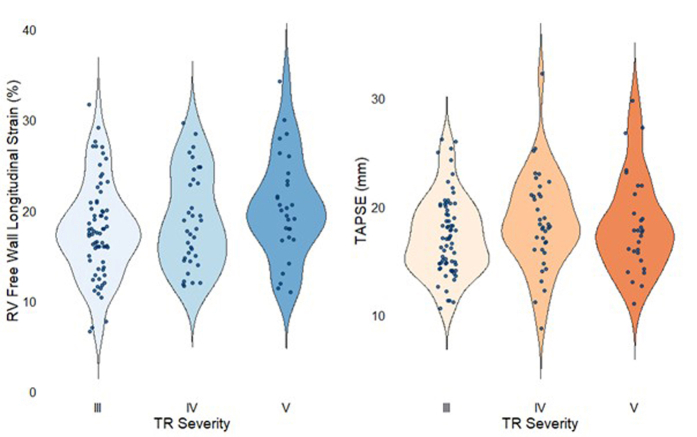


In vendor A, RVFWS demonstrated an inverse correlation with PASP (Spearman’s ρ = –0.298, *p* < 0.001, Figure 5) and a positive correlation with TR severity (ρ = 0.174, *p* = 0.042, Supplementary Figure 3). In contrast, TAPSE correlated with neither PASP (ρ = –0.123, *p* = 0.154) nor TR grade (ρ = 0.129, *p* = 0.133). In vendor B, no associations were observed between either functional parameter and PASP or TR severity (Figure 5 and Supplementary Figure 4).

## Discussion

This multicenter study is the largest to date evaluating the prognostic value of RVFWS- and TAPSE-derived RV-PA coupling in patients undergoing TTVr, and the first to compare these indices across two widely used vendor-specific strain analysis platforms. The principal findings can be summarized as follows.

1. ROC-derived cutoff values for each parameter identified patient subgroups with markedly different 2-year outcomes, confirming their clinical applicability as measures of RV-PA coupling.
2. TAPSE/PASP emerged as the more robust RV-PA coupling index compared to RVFWS/PASP and was independently associated with the composite endpoint of HFH-free survival at 2 years in both cohorts.
3. Both coupling indices demonstrated only modest discriminative performance, with RVFWS values showing pronounced variability between vendors and cohorts, yet retaining consistent performance across different strain analysis platforms.

Our findings extend prior work on RV-PA coupling in patients with TR. TAPSE/PASP has been widely validated as a prognostic marker across various cardiovascular conditions, including left- and right-sided valve disease, pulmonary hypertension, and heart failure.^2^^,^^16^, ^17^, ^18^ By contrast, although the body of evidence for RVFWS/PASP is expanding, its prognostic value in patients with TR, particularly after TTVr, remains less well defined. In conservatively managed TR, RVFWS and RVFWS/PASP, but not TAPSE or TAPSE/PASP, were independently associated with adverse outcomes.^6^^,^^8^ These prior findings provided the rationale for our hypothesis that RVFWS/PASP might offer incremental prognostic value in patients undergoing TTVr.

Contrary to this expectation, RVFWS/PASP did not outperform TAPSE/PASP in either of our vendor-defined cohorts. The robustness of this finding is supported by its consistency across two clinically distinct populations, differing not only in strain analysis platform but also in baseline characteristics, disease severity, and procedural modality. In fact, TAPSE/PASP remained independently associated with survival free from HFH at 2 years in both cohorts, whereas RVFWS/PASP was predictive only in the vendor A cohort. In vendor B, absolute RVFWS values fell within the normal reference range^13^ despite patients presenting with more advanced RV remodeling and higher event rates compared with vendor A. This paradox may be explained either by vendor-related differences in strain assessment or by overestimated strain values in the setting of severe TR. Intervendor variability in deformation imaging is a well-recognized challenge: despite recent efforts by the European Association of Cardiovascular Imaging/American Society of Echocardiography/Industry Task Force to standardize strain analysis, intervendor bias persists and may contribute to discrepant findings across cohorts.^19^ Although a pragmatic cutoff of 20% is widely accepted to distinguish normal from abnormal RVFWS irrespective of vendor, truly vendor-specific thresholds are lacking, underscoring the methodological challenges of comparing absolute strain values across platforms.^5^

Nevertheless, ROC analysis identified cutoffs for each coupling parameter that resulted in patient subgroups with clinically meaningful differences in 2-year outcomes in both cohorts. Notably, all cutoffs (vendor A: RVFWS 0.388%/mmHg, TAPSE 0.367 mm/mmHg: vendor B: TAPSE 0.442 mm/mmHg) closely corresponded to previously reported thresholds,^2^^,^^8^^,^^20^ with the exception of the relatively high cutoff for RVFWS/PASP in vendor B with 0.583%/mmHg, again underlining its suboptimal performance in this cohort.

Importantly, the overall discriminatory ability of both parameters proved robust across sensitivity analyses: results remained stable irrespective of procedural modality or whether PASP was derived invasively or estimated echocardiographically, with no difference between the two parameters. Notably, indices based on PASP_inv_ yielded numerically higher discriminative performance than those based on estimated PASP, consistent with prior reports showing that echocardiography tends to underestimate pulmonary pressures in advanced TR and invasive RV-PA coupling provides superior prognostic stratification in this setting.^21^ In addition, estimated PASP was in general lower than PASP_inv_ in vendor B, which likely contributed to the observed differences in coupling ratios and may have attenuated the overall prognostic performance of both indices in vendor B (vendor A: RVFWS/PASP 0.621, TAPSE/PASP 0.646; vendor B: RVFWS/PASP 0.564, TAPSE/PASP 0.599). In line with this, the use of PASP_inv_ resulted in a modest improvement in discriminatory performance in vendor B, indicating partial recovery when pressure estimation is corrected, yet without achieving the performance observed in vendor A. Bland–Altman plots indeed confirmed minimal bias between estimated and invasively measured PASP in vendor A, but substantial underestimation in vendor B.

We anticipated RVFWS/PASP to provide superior risk stratification, reflecting the higher sensitivity of RV strain to incipient RV dysfunction.^6^ Mechanistically, RVFWS showed a weak, inverse correlation with PASP and a weak, positive correlation with TR severity, potentially indicating greater susceptibility to hemodynamic loading conditions, whereas no such associations were found for TAPSE. These observations, however, could not be confirmed in vendor B and should therefore be regarded as hypothesis-generating and interpreted with caution regarding potential clinical implications. In this cohort, RVFWS values clustered within a relatively narrow range and did not vary meaningfully with PASP, suggesting that a combination of intervendor variability and population-related differences may have contributed to the attenuated prognostic performance of RVFWS/PASP in this cohort. In this context, it is noteworthy that the key reference study on TAPSE/PASP in severe TR likewise demonstrated that TAPSE was not correlated with PASP, underscoring its robustness as a prognostic marker.^2^ Discriminative ability in that cohort was comparable to ours with an area under the curve of 0.632, and the median TAPSE/PASP ratio (0.406 mm/mmHg) closely approximated the thresholds identified in both of our vendor-specific cohorts. Baseline characteristics were broadly similar with respect to age, TAPSE, PASP, TR severity, and functional status; however, patients in the TriValve Registry may have represented a higher-risk population with more frequent left ventricular systolic dysfunction and a greater surgical risk profile, which may partially explain differences in absolute risk stratification patterns. Accordingly, when interpreting load-dependent parameters of RV function, integration with PASP appears essential for a more accurate characterization of RV performance. This is supported by recent evidence demonstrating that preserved RVFWS predicted favorable outcomes only in patients without severe TR or pulmonary hypertension, whereas integration with PASP retained prognostic relevance across all disease stages.^22^

A recent study in conservatively managed patients with severe TR reported a markedly lower optimal RVFWS/PASP cutoff of 0.26%/mmHg with higher discriminatory ability (area under the curve 0.74), despite median coupling values (≈0.40 for both RVFWS/PASP and TAPSE/PASP) being similar to those observed in our study.^8^ Differences in population characteristics may account for this variability: the cohort consisted largely of noninterventional patients with slightly better functional status, comparable RV, and left ventricular dimensions including TAPSE, higher PASP values, and a substantial proportion with prior cardiac surgery. Interestingly, RVFWS/PASP—but not TAPSE/PASP—was independently associated with all-cause mortality, although the limited number of events (N = 16) reduces statistical power and may also contribute to divergent prognostic behavior compared with our interventional TTVr population.

In our study, however, RVFWS/PASP did not provide incremental prognostic value beyond TAPSE/PASP. Whether this reflects the distinct physiological behavior of the two indices—with RVFWS being more sensitive in earlier disease stages but less reliable in advanced remodeling—or rather methodological aspects of acquisition, given that TAPSE is easily measurable in most patients, whereas strain analysis requires optimal image quality, or a combination of both factors, cannot be determined from the present data.

Despite substantial symptomatic and anatomical improvement after TTVr—with more than half of patients improving by at least 1 NYHA class and residual TR ≤ 2+ achieved in a substantial percentage of patients—two-year event rates remained high at 34 and 38%. This persistent residual risk underscores the need for robust predictors of outcome beyond procedural success and supports continued efforts to refine RV-PA coupling indices for clinical risk stratification. Importantly, whether RVFWS/PASP provides incremental prognostic information in patients with less advanced disease stages remains uncertain and warrants clarification in future prospective studies.

### Strengths and Limitations

This study has several strengths. It represents the largest multicenter analysis to date evaluating strain-based RV-PA coupling in patients undergoing TTVr and the first to directly compare RVFWS- and TAPSE-derived indices across two independent vendor platforms and cohorts with long-term follow-up. Echocardiographic data were analyzed with vendor-specific software by experienced echocardiographers blinded to outcomes. The inclusion of two clinically distinct cohorts, together with consistent discriminatory results across multiple sensitivity analyses, enhances both the robustness and generalizability of our findings.

Several limitations must also be acknowledged. First, the retrospective observational design limits causal inference and carries an inherent risk of selection bias. Second, no echocardiographic core laboratory adjudication was performed, and intervendor differences in strain quantification may have influenced absolute values. Finally, the pronounced baseline and procedural differences between cohorts, although supportive of generalizability, may at the same time have contributed to discrepant results and limit direct comparability.

## Conclusion

In this multicenter study of patients undergoing TTVr, the discriminatory performance of RV-PA coupling indices was similar between different vendors and cohorts. TAPSE/PASP was independently associated with long-term outcomes in both cohorts, whereas this held true for RVFWS/PASP only in 1 cohort. Although RVFWS/PASP did not provide incremental prognostic value in this advanced TR population, it may be beneficial in patients at earlier disease stages. As strain imaging continues to evolve and is increasingly adopted in clinical practice, clarifying its incremental prognostic value in patients with less advanced TR remains an important objective for future prospective studies.

## Ethics Statement

This study was conducted in accordance with the Declaration of Helsinki and institutional ethical standards. Approval was obtained from the local ethics committees of all participating centers. Because this was a retrospective analysis using anonymized clinical and imaging data, the requirement for written informed consent was waived.

## Funding

The authors have no funding to report.

## Disclosure Statement

Jennifer von Stein has received speaker honoraria and travel expenses by 10.13039/100006520Edwards Lifesciences. Lukas Stolz has received speaker honoraria from 10.13039/100006520Edwards Lifesciences. Michael I. Brener is supported by 1K23HL177311-01A1 and has received grant funding from 10.13039/100011949Abbott Vascular and 10.13039/100004331J&J/10.13039/100020297Abiomed, as well as consulting honoraria from CroiValve and Ventricord. Maria Isabel Körber has received travel support by JenaValve and lecture fees from 10.13039/100006520Edwards Lifesciences and 10.13039/100000046Abbott. Stephan Baldus has received honorarium for consultation by 10.13039/100000046Abbott and 10.13039/100006520Edwards Lifesciences. Jörg Hausleiter received research support and speaker honoraria from 10.13039/100006520Edwards Lifesciences. Roman Pfister has received speaker fees by 10.13039/100006520Edwards Lifesciences and 10.13039/100000046Abbott. Christos Iliadis has received travel support by 10.13039/100000046Abbott and 10.13039/100006520Edwards Lifesciences and consultant honoraria by 10.13039/100000046Abbott and 10.13039/100006520Edwards Lifesciences.

The other authors had no conflicts to declare.

## Figures and Tables

**Supplementary Figure 4:**